# Adolescent alcohol binge drinking and withdrawal: behavioural, brain GFAP-positive astrocytes and acute methamphetamine effects in adult female rats

**DOI:** 10.1007/s00213-024-06580-2

**Published:** 2024-05-06

**Authors:** Priscila A. Costa, Nicholas A. Everett, Anita J. Turner, Laísa S. Umpierrez, Sarah J. Baracz, Jennifer L. Cornish

**Affiliations:** Behavioural Neuropharmacology Laboratory, School of Psychological Sciences, Faculty of Medicine, Health and Human Sciences, Macquarie University, North Ryde, Sydney, NSW 2109 Australia

**Keywords:** Alcohol, Adolescence, Sucrose, Anxiety-like behaviour, Methamphetamine, Astrocytes

## Abstract

**Rationale:**

Alcopop beverages are generally the first alcoholic beverage that young females drink which contain high levels of sugar and alcohol. The over-consumption of these drinks may encourage alcohol co-administration with methamphetamine (METH) impacting on drinking behaviour and glial function.

**Aims:**

The aims of this study were to evaluate the effect of adolescent binge alcohol exposure on consumption level, anxiety-like behaviour, cross-sensitization with METH and on astrocyte expression in reward related brain regions.

**Methods:**

Adolescent female Sprague-Dawley rats had daily 1-hour oral alcohol consumption of alcopop (ALCP; with sucrose) or ethanol-only (ETOH; without sucrose), transitioned from 5 to 15% (v/v) ethanol content for 34 days. Water and sucrose groups act as controls. During alcohol withdrawal, rats were tested for anxiety on the elevated plus maze (EPM) and locomotor activity following saline or METH (1 mg/kg i.p) treatment. Brains were then collected to assess astrocyte immunofluorescence for glial fibrillary acidic protein (GFAP) in reward-related brain regions.

**Results:**

Rats pretreated with 5% ALCP consumed significantly more volume and ethanol intake when compared to 5% EtOH rats. Both ALCP and EtOH groups had a higher preference ratio for 5% than 15% alcohol solutions and ALCP rats had greater ethanol intake at 15% than EtOH rats. Alcohol withdrawal showed no significant differences between groups on anxiety, METH cross-sensitization effects or GFAP intensity in the regions studied.

**Conclusions:**

Overall, the addition of sucrose to alcoholic solutions encouraged female rats to consume larger volumes and greater ethanol intake compared to ethanol-only solutions, yet did not have long lasting effects on behaviour and astrocytes.

## Introduction

Alcohol binge drinking is characterized by having 4 or more standard drinks by women, and 5 or more drinks by men in a single event over approximately 2 h (NIAAA, 2017), and it has been reported that 7% of young Australians, aged 12–17 years old, consume 11 or more standard drinks on a single drinking occasion (AIHW 2017). Indeed, early consumption of alcohol in life has also been associated with greater risk of injury, drug experimentation, changes to brain development and the development of depression (Bonomo et al. 2004; Townshend and Duka 2005; Luciana et al. 2013). Early alcohol consumption by teenagers is a moderate predictor of alcohol dependence in adulthood (Bonomo et al. 2004; see review (Metzner and Kraus 2008), where early heavy alcohol consumption is the highest risk indicator for developing an alcohol dependency later in life (Metzner and Kraus 2008). Early alcohol use may also be associated with other substance use, such as methamphetamine (METH) which is commonly co-administered with alcohol (Bonomo et al. 2004; Luciana et al. 2013).

Alcoholic drink consumption typically includes spirits, beer and wine, however premixed spirits or “alcopops” are the most commonly consumed alcoholic beverage in young adults in Australia (Rossheim and Thombs 2017), particularly amongst females (Bridle et al. 2012). According to the AIHW (2017), 40% of young drinkers prefer alcopops, in which the taste, alcohol strength and the cost are some factors that may influence this preference for alcopops among the youth (Jones and Reis 2011). Alcopop beverages are well known for their high levels of both sugar and alcohol. The addition of sugar to these drinks has been reported to increase the appeal of alcohol to young adults as the alcoholic taste is masked and becomes more palatable (Roberts et al. 1999). The reinforcing effects of the sweet taste are associated with activation of the same reward-related neurotransmitter systems affected by abused drugs, including alcohol, such as dopamine and serotonin (Fortuna 2010). As such, activation of these neurotransmitter systems increases the desire to drink alcopops.

Alcohol binge drinking has also been associated with many health conditions, and anxiety disorders are common (Lee et al. 2020). Anxiety has often been associated with alcohol drinking and withdrawal (Doremus and Spear 2007). In preclinical studies, chronic alcohol exposure by two-bottle choice (water and 10% ethanol) in the homecage (7 months) increased anxiety-like behaviour at 1-week of withdrawal in young adult female mice (Xu et al. 2021), and a recent study has shown that repeated cycles of binge-drinking of ethanol by gastric gavage during adolescence (> 20 days) produced long-lasting anxiety into adulthood after withdrawal in female rats (Queiroz et al., 2022). However, the effect of alcopop binge drinking on anxiety following withdrawal has yet to be reported.

Furr et al. (2000) showed that frequent alcohol drinkers are five times more likely to use METH when compared to non-drinkers, and this association is greater when alcohol is taken in a binge drinking pattern (Bujarski et al. 2014). Indeed, Fultz and Szumlinski (2018) found that binge drinking alcohol increased risk for METH co-abuse in male mice. Despite some clinical and pre-clinical evidence of alcohol-METH interaction, less is known about the neurobiological mechanisms that drive these effects.

Ethanol is a well known substance that markedly impacts the function of numerous neurons and neurotransmitters in the nervous system, however recent research has focused on how ethanol affects the glial cells, particularly astrocytes, changing their function and morphology. Astrocytes are the most abundant glial cells in the central nervous system and play an essential role in brain communication and plasticity among glial cells and the neurons that surround them (Miguel-Hidalgo 2009; Volterra and Meldolesi 2005). Erickson and colleagues (2021) demonstrated that ethanol consumption regulated calcium signaling by astrocytes in male mice, that may contribute to early behavioural regulation and the development of alcohol use disorders. However, further work needs to be conducted to understand the involvement of astrocytes in the behavioural effects of alcohol, particularly in females.

These gaps in the literature emphasize a need for a better understanding of the effect of adolescent alcohol exposure on anxiety and future use of METH, as measured here by behavioural cross-sensitization, in addition to measures of the effect of ethanol use on astrocytes in reward related brain regions. Therefore, the present study used an alcopop binge drinking model during the adolescence period of female rats and analysed their anxiety levels, locomotor activity after acute METH exposure following alcohol withdrawal, as well as assessing the possible inflammatory effects of alcohol binge drinking on the density of astrocytes in reward-related brain regions 2-weeks after alcohol withdrawal.

## Methods

### Animals

Sixty-six adolescent female Sprague Dawley rats at postnatal day (PND) 21 were obtained from pregnant dams purchased from Animal Resources Centre (ARC, Perth, Australia). After weaning, rats were housed in groups of 3–4 under a 12 h light-cycle (lights on at 6am) in open top cages (64 × 40 × 22 cm) filled with corn cob bedding, plastic tunnel and wooden sticks. Food and water were available *ad libitum*, except when they had mild water restriction while performing the oral consumption phase. All experimental procedures were approved by the Macquarie University Animal Ethics Committee (Animal Research Authority 2018-021) in accordance with the Australian Code of Practice for the Care and Use of Animals for Scientific Purposes (8th Edition, 2013).

### Drugs and solutions

Ethanol (EtOH; 99.5% v/v) was purchased from Sigma-Aldrich (Australia). Sucrose was obtained from Colonial Sugar Refine Company (CSR, Australia); Methamphetamine hydrochloride (METH) was purchased from the Australian Government Analytical Laboratories (Australia). The alcoholic solutions were prepared every second day using the formula [V1 × M1 = V2 × M2] and density of EtOH 0.79 g/cm^3^, adding EtOH in distilled water in a concentration of 5 or 15% ethanol. For alcopop solutions 10% of sucrose was added. For intraperitoneal (ip) injection, METH was prepared in physiological saline (0.9% NaCl; 1 ml/kg).

### Apparatus

Adolescent rats (PND23) were individually placed in metabolic cages (Tecniplast, NSW, Australia; cylindric 20 cm (d) × 15 cm (h)) daily for a 1-hour oral consumption session. The day prior to the start of sessions they were acclimatized to the cages to avoid the potential confound of stress on drinking behaviour. Each metabolic cage provided access to a single bottle of solution (by group) and a container to control for spillage under each bottle. Each rat was allocated to the same cage for the duration of the experimental sessions. Bottled solutions were weighed daily before and after each session, with the weight of fluid in the spillage container subtracted from the total amount of fluid consumed by each rat.

### Estrous cycle

Vaginal smears were collected to determine whether the hormonal phase had an impact in alcohol consumption only after performing the Preference Test (PT) day (see *Experimental procedure*). Estrous cycle stage was assessed according to Souza and colleagues (2014), by examining the cellular characteristics from the vaginal smears collected with a plastic micro-pipette containing ambient temperature physiological saline, gently inserting into the vaginal canal. After a few seconds, the pipette was then inserted again to collect the sample with cells and transferred to slides, then observed under a light microscope (10 and 40 × objective lenses). Briefly, the leucocytes, cornified epithelial cells and nucleated epithelial cells were considered for this analysis, where the four phases of estrous cycle were based according to the proportion of these cells: (1) proestrus, (2) estrus, (3) metestrus, (4) diestrus (Ajayi and Akhigbe 2020).

### Elevated plus maze

The elevated plus maze (EPM) was used at 1 week withdrawal from oral sessions to measure anxiety-like behaviours such as fear of open spaces, but also provided measures of exploration and risk assessments while in the apparatus. Although past research has shown withdrawal effects of alcohol to induce anxiety in first 24–48 h (Gonzaga et al. 2016; Patkar et al. 2019), a one-week withdrawal period was chosen here to investigate a persistent anxiety phenotype following chronic alcohol use. The plus-shaped platform was made of opaque grey plastic (Perspex), elevated 50 cm above the floor, with two open arms and two closed arms measured 50 cm (length) × 10 cm (width), and the closed arms surrounded by a wall of 40 cm in height. Rats were placed in the centre of the maze facing an open arm and allowed to explore the maze for 5 min, as previously described by our group (Baracz et al. 2022). Measures included percentage of time spent on the open arms (% Open  =  (time spent open arms/(time spent open arms + time spent closed arms) ×100), number of entries in the open arm, closed arms and number of crossing the centre square; as well the time spent in each part of the maze. These results were further investigated by scoring ethologically relevant behaviours such as rearing, grooming and risk assessment behaviours using the software Behavsoft® (Costa et al. 2019). Rearing was defined as standing on the hind legs; grooming was scored when the animal was licking and cleaning its body; head dipping was scored when the rats head was pointed downward over the edge of the platform while in open arm; and stretch attend posture was defined as stretching the head forward to the open arm without stepping forward into the open arm. Protected behaviours describe those performed in the closed arms, with open behaviour defined as behaviours conducted on the open arms (Carobrez and Bertoglio 2005).

### Locomotor activity

**Fig. 1:**
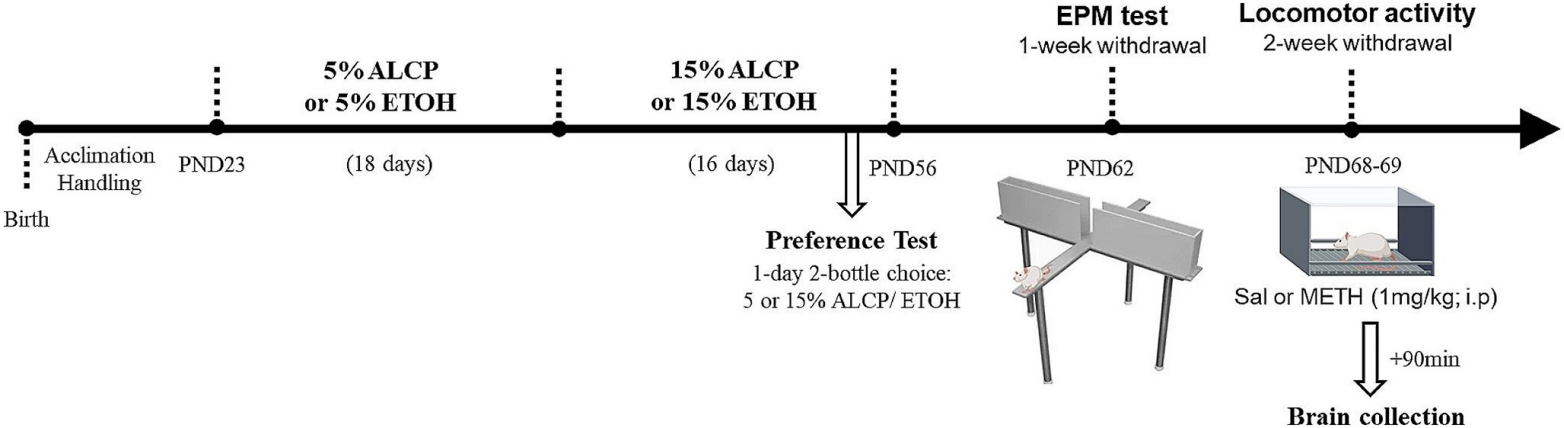
Experimental procedure timeline. PND, postnatal day; ALCP, alcopop; ETOH, ethanol; EPM, elevated plus-maze test; METH, methamphetamine; SAL, saline. Adapted from Biorender©

To investigate the development of behavioural cross-sensitization from repeated alcohol consumption to METH-induced behaviour, locomotor activity was recorded at 2 weeks of withdrawal from oral sessions (Fig. 1). Locomotor activity was measured in Actimeter infrared chambers (L 36 × W 24 × H 19 cm; Imetronic Pessac, France) following a protocol well established in our laboratory (Wearne et al. 2015; Umpierrez et al. 2022). Each chamber consisted of a removable plastic box with mesh wire top and flooring. Within each chamber were four parallel horizontal infrared sensors which recorded photocell beam breaks in three dimensions. Total locomotor activity was calculated by summing the total horizontal activity for each rat (sum of activity in the front of the box, activity in the back of the box, and movement between the front and back). Prior to all testing sessions, rats were placed in the test chamber for 15 min to reduce novelty-induced increases in activity before locomotor activity was recorded (1 h). Each chamber was cleaned with F10 veterinary disinfectant spray solution (F10®Produts; Health and Hygiene, South Africa) between trials.

### Experimental procedure

Rats were randomly separated into 4 experimental groups in this study: water (H2O), 10% sucrose (SUC), ethanol-only (ETOH) and alcopop (ethanol + 10% sucrose; ALCP). Only ETOH and ALCP groups had ethanol content in their solutions (increased from 5 to 15% v/v on D19). Prior to commencing the experiment, mild water restriction was applied to all groups, where rats had 4-hours access to water every day 30 min after each drinking session. All animals were placed into individual cages for 1-hour daily drinking sessions during their adolescence period (PND23-56). The ETOH and ALCP groups were exposed to a single bottle with 5% of ethanol for 18 days and to 15% of ethanol for 15 days (Fig. 1). They were also tested for an alcohol PT 2 days prior to alcohol withdrawal, where 2 bottles of 5 and 15% of ethanol-only or alcopop solution (side counterbalanced across each group) were presented to each cage and the consumption from each bottle was evaluated for 1 h. Then, immediately after the PT the estrous cycle of each animal was identified. After the oral consumption period, all animals were placed into a 14-day alcohol withdrawal period in their home cages with water and food *ad libitum*.

To avoid any signs of alcohol hangover state in the first hours post-deprivation of alcohol (Karadayian et al. 2013) as well the levels of alcohol left in the body system that can last for several days after cessation (Tice et al. 2022), the EPM test was performed 1-week alcohol withdrawal. After their first week on alcohol withdrawal the rats were exposed to the EPM test for 5 min/each.

As alcohol users have a higher risk of METH use (Furr et al. 2000), the impact of their interaction is important to determine. Animal studies showed sensitization to ethanol alone (Zapata et al. 2006 or to METH alone (Wearne et al. 2015) after 14 days of withdrawal, however little information exists about their interaction on behaviour. In addition, there is limited information on the impact of prior alcohol exposure on subsequent METH use and relapse, although a study showed that past alcohol use reduced cue-primed METH seeking in female rats (Kline and Yamamoto 2022). In order to align with this study and those using sensitization paradigms, we tested cross-sensitization between ethanol and METH following a 2-week alcohol withdrawal period, they were placed into the locomotor activity apparatus after a saline or METH ip injection (1 mg/kg) and measured for 1 h. All experimental procedures occurred at light-cycle.

### Tissue collection and immunofluorescence

Ninety minutes following each saline or METH injection, rats were deeply anaesthetised with lethobarb injection (135 mg/ml sodium pentobarbitone, diluted 1:4 in saline (81.25 mg/ml), 1.5 ml, and an intracardiac perfusion was conducted with 250 ml ice-cold heparinized saline (10 IU/ml) followed by 250 ml ice-cold 4% paraformaldehyde (PFA) to fix the brain tissue. For the purpose of this study, only the animals which received prior saline injection were analysed. Brains were removed and further fixed overnight in 4% PFA at 4 °C, then immersed in cryoprotectant solution (30% ethylene glycol, 30% sucrose, 2% polyvinylpyrrolidone dissolved in 0.1 mol L-1 PBS) and stored at -20 °C prior to sectioning.

Coronal brain Sect. (50 μm) were sliced using vibratome (VT1200S, Leica Microsystems, Australia) in a 1:4 sequential series of free-floating sections and stored in cryoprotectant solution at -20 °C until analysis was conducted. The immunofluorescence procedure was based on previous work by our group (Baracz et al. 2020) and used to identify glial fibrillary acidic protein (GFAP) to evaluate the density of astrocyte cells in the brain. First, tissue sections were washed three times for 30 min in Tris phosphate-buffered saline (TPBS; Tris-HCL 10 mM + sodium phosphate buffer 0.1 M + 0.9% NaCl), followed by a 30-min wash in Tris (10 mM, pH 10) + Tween 20 (0.01%) at 80 °C to enhance antigen retrieval. The tissue was then left to cool for at least 1 h before three 5-min washes in TPBS. The tissue sections were then pre-incubated with TPBSm (TPBS with 0.05% merthiolate) and 10% normal horse serum for 2 h for blocking against non-specific binding, and after the tissue sections were incubated with primary antibody for 6–8 h at room temperature and 40–42 h at 4 °C. The primary antibody used for detecting the astrocytes was a polyclonal goat anti-GFAP (dilution 1:1000; ab53554; Abcam, USA). After three 30-min post-primary antibody washes, sections were again pre-incubated in TPBSm and 2% normal horse serum for 1.5 h before incubation with fluorescently conjugated secondary antibodies (dilution 1:500; A11055; Alexa488-conjugated Donkey anti-goat; Jackson ImmunoResearch, West Grove, PA, USA) overnight at 4 °C. After washing off the secondary antibody, sections were mounted on glass slides and cover slipped with mounting medium (Dako, Glostrup, Denmark), which were then viewed on a fluorescence microscope. A control experiment to test specificity of the markers was performed by omitting the primary or secondary antibodies to confirm the absence of immunostaining in brain tissue.

### Imaging and intensity analysis

**Fig. 2:**
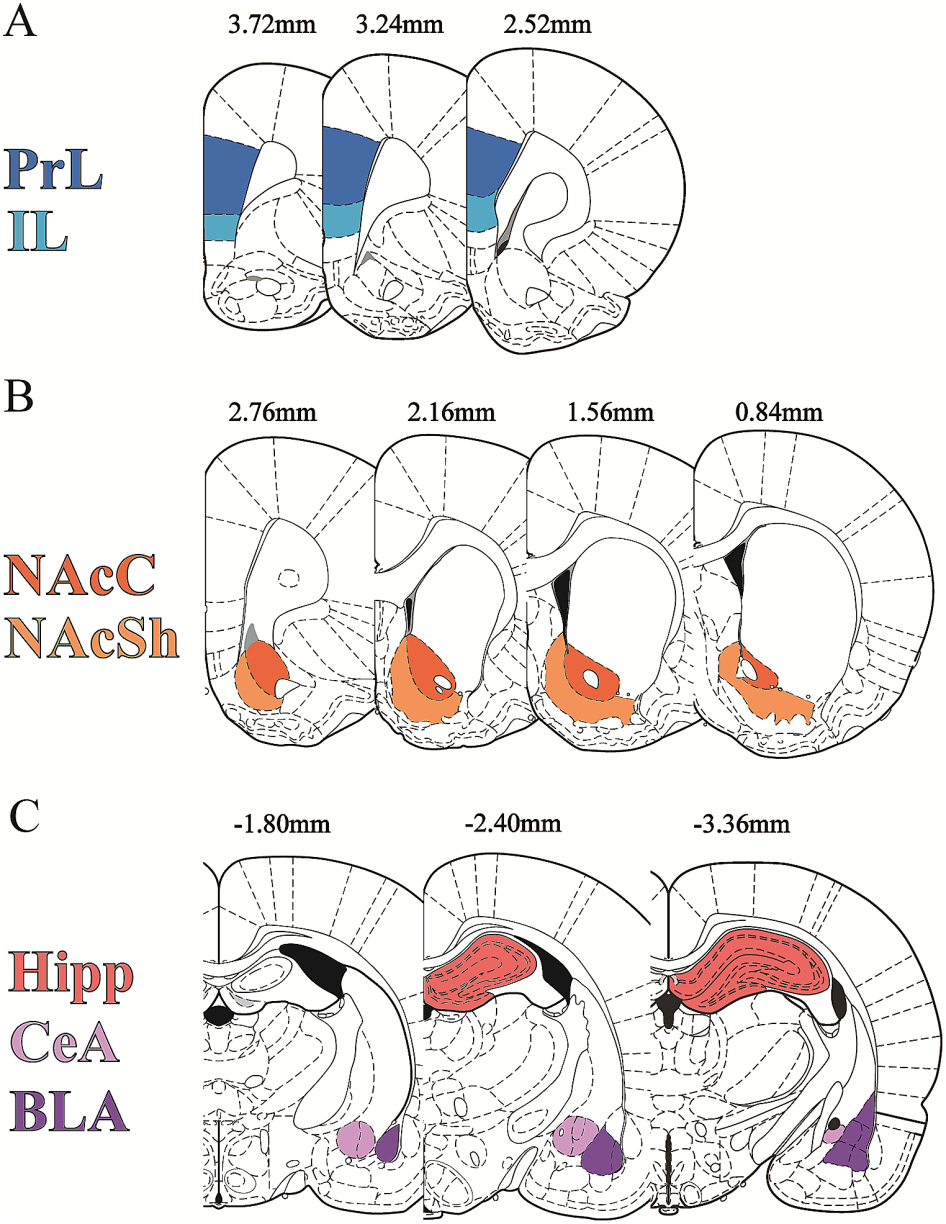
Representation of anatomical coronal diagrams depicting each brain region of interest and the coordinates (mm from Bregma) at which the regions were analysed. (**A**) Ranging of bregma levels containing prelimbic (PrL) and infralimbic (IL) cortices; (**B**) Ranging of bregma levels containing nucleus accumbens core (NAcC) and shell (NAcSh); (**C**) Ranging of bregma levels containing hippocampus (CA1, CA3, dendate gyrus -DG), central amygdala (CeA) and basolateral amygdala (BLA). Adapted from the Rat Brain Atlas (Paxinos and Watson 2007)

Images were acquired and analysed across a total of 13 unilateral brain slices per animal, and a total of nine subregions of interest (ROIs) were analysed using a range of equidistant coordinates of each brain region. Prelimbic (PrL), infralimbic (IL), nucleus accumbens core (NAcC) and shell (NAcSh), hippocampus subregions (CA1, CA3) and dendate gyrus (DG), central amygdala (CeA) and basolateral amygdala (BLA) were analysed using fluorescence microscope (Axioimager Z3; Carl Zeiss, Oberkochen, Germany) ranging from **+** 3.72 to − 3.36 mm from bregma displayed in Fig. 2. The images were acquired by 20x magnification, 1024 × 1024, tile scanning for each ROI, and all settings were maintained within all sections during image acquisition. Brightness/contrast and pixels were the same for all images shown per figures. Analysis of integrated density was performed blind to treatment groups using the imaging software Fiji-ImageJ (Schindelin et al. 2012). The fluorescence integrated density for each brain region was calculated based on the corrected total cell fluorescence of each ROI as using the equation [CTCF = raw integrated density of ROI – (Area of ROI × Mean fluorescence value of background readings)], available at [https://theolb.readthedocs.io/en/latest/imaging/measuring-cell-fluorescence-using-imagej.html] and modified from (Ituarte et al. 2016).

### Statistical analysis

Statistical analyses were performed using SigmaPlot 12.0 and displayed by Prism 9 software (GraphPad, San Diego, CA). For testing our hypothesis, two-way ANOVA repeated measures (RM) was used to analyse the oral consumption and the alcohol content comparing groups and the different days they were exposed. EPM, locomotor activity and GFAP intensity data were analysed using one-way ANOVA to compare all 4 groups. Significant main effects and/or interactions were followed by post-hoc Tukey test and *P* < 0.05 was considered as statistically significant. Data were reported as mean ± standard error of the mean (SEM).

## Results

### Oral consumption and ethanol intake

**Fig. 3:**
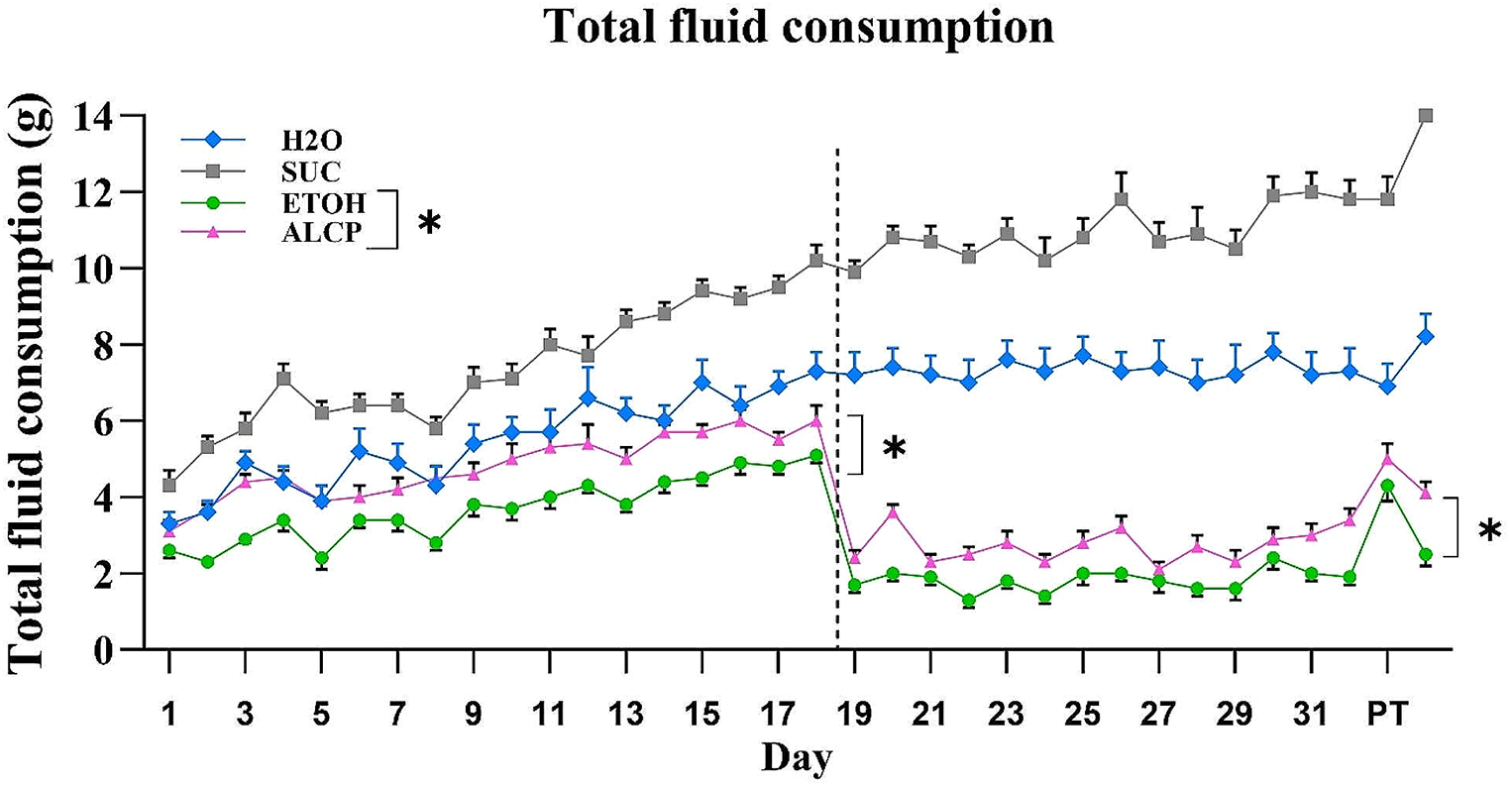
Total fluid consumption. H_2_O, water control (blue); SUC, sucrose (grey); ETOH, ethanol (green); ALCP alcopop (pink); PT, preference test day (day 33). The ethanol content for either ALCP or ETOH groups was 5% (from day 1–18) and 15% (from day 19–34). Data are expressed as mean ± (SEM) and significant difference as a main effect of group or dose when *P < 0.001

The total fluid consumed by rats during their daily session in the adolescent period was assessed and the profiles of oral consumption from each group are displayed in Fig. 3. Considering each phase of concentration of solution (5 and 15%), all animals increased their fluid intake overtime (except H2O group which was steady for most of the days across time) with only SUC group significantly increasing their own fluid consumption only from day 19–34 compared to the other groups (*F*(1,99) = 17.927; *P* < 0.001) consuming alcoholic solution at the higher concentration of 15% (although no different solutions were given to this control groups). Within the alcoholic solutions ALCP and ETOH, two-way RM ANOVA detected no significant interaction by group × dose (*F*(1,33) = 0.239; *P* = 0.628), after a posthoc Tukey test a main effect of both group (*F*(1,33) = 31.964; *P < 0.001) and dose (*F*(1,33) = 77.196; *P < 0.001) were identified where ALCP rats having greater total of fluid consumption compared with ETOH rats in both alcohol concentrations. The body weight was not significantly different across groups, having an average range from 181 to 192 g of body weight in the last oral drinking session (Day 34).

**Fig. 4:**
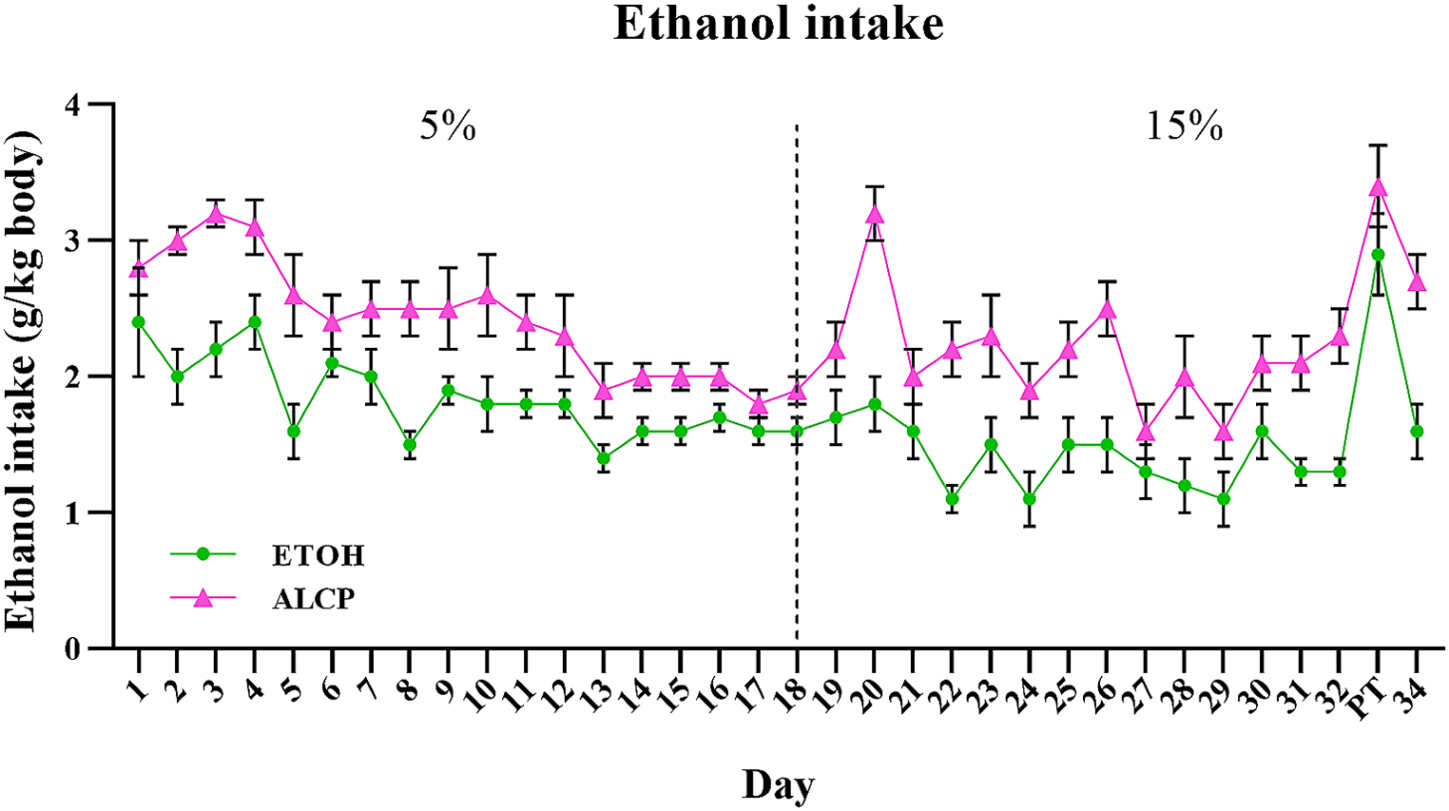
Total daily ethanol intake by ALCP and ETOH rats. ETOH, ethanol (green); ALCP, alcopop (pink); PT, preference test day (day 33). The ethanol content for either ALCP or ETOH groups was 5% (from day 1–18) and 15% (from day 19–34). Data are expressed as mean ± (SEM)

In order to analyse ethanol intake (Fig. 4), two-way RM ANOVA was used and no interaction was found between group × dose (*F*(1,33) = 2.751; *P* = 0.107), but a main effect of group was detected (*F*(1,33) = 4.807; *P* = 0.035) and no effect on dose (*F*(1,33) = 0.017; *P* = 0.896), with ALCP having greater ethanol intake than ETOH group regardless of alcohol concentration. The female rats displayed a range of ethanol intake between 1.1 and 3.4 ± 0.3 g/kg/h (with highest amount by ALCP group) across time, amount considered as moderate to high ethanol intake levels overtime, relevant to induce binge intoxication (Holgate et al. 2017).

### Alcohol preference test

**Fig. 5:**
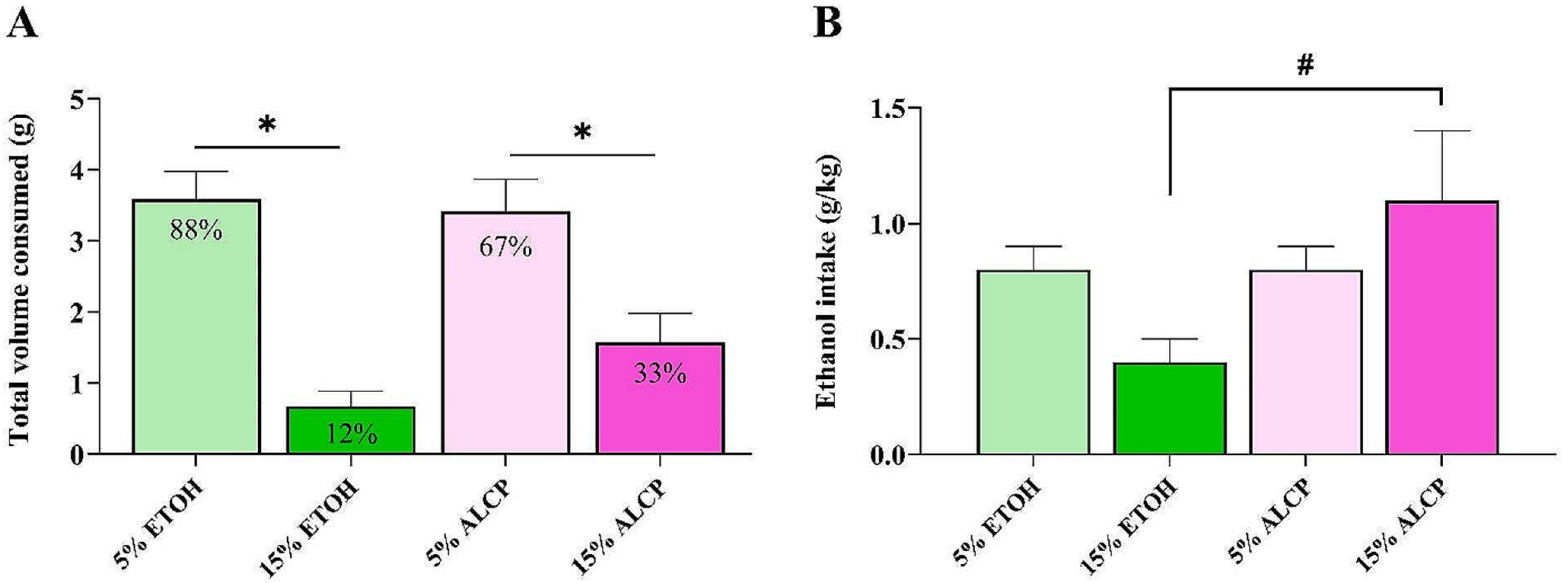
Preference Test Day. (**A**) Total volume consumed and preference ratio; (**B**) Total ethanol intake of each ethanol content. ETOH, ethanol (green); ALCP, alcopop (pink). Data displayed as mean ± SEM, and significant difference when *P* < 0.05 (* compared within group; # compared between groups). ETOH (ethanol, green); ALCP (alcopop, pink)

The PT displays a sum of the total fluid intake from both bottles of 5 and 15% content offered to each rat from ALCP and ETOH. Two bottle choices were given to the rats for testing preference to the alcoholic concentration between 5 and 15% in each group (Fig. 5). The majority of EtOH and ALCP rats preferred the solution with the low alcohol content 5% EtOH (88%) or 5% ALCP (67%) instead of 15% alcohol solutions (total *n* = 17–18 rats/group). Two-way RM ANOVA was used and there was no significant interaction between group × dose *F*(1,33) = 1.323; *P* = 0.258, however a main effect of dose *F*(1.33) = 26.277; *P < 0.001, as the low dose was significant different from the high dose from both ETOH (*P* < 0.001) and ALCP (*P* = 0.008) groups (Fig. 5A). When analyzing the ethanol intake (g/kg), no interaction was found for group × dose (*F*(1, 33) = 2.751; *P* = 0.107), however a main effect was detected by group (*F*(1, 33) = 4.807; *P* = 0.035) where ALCP had a similar amount of ethanol intake (g/kg) compared with ETOH group within low dose (*P* = 0.903), but ALCP group had significant higher amount of ethanol within 15% solution compared to ETOH rats (#*P* = 0.012; Fig. 5B). Thus, confirming our hypothesis that the addition of sucrose in alcoholic concentration (ALCP) makes higher concentration of alcohol (15%) more appealing by female rats to encourage higher ethanol intake, although the lower percentage alcohol solution was preferred overall.

### Estrous cycle

**Fig. 6:**
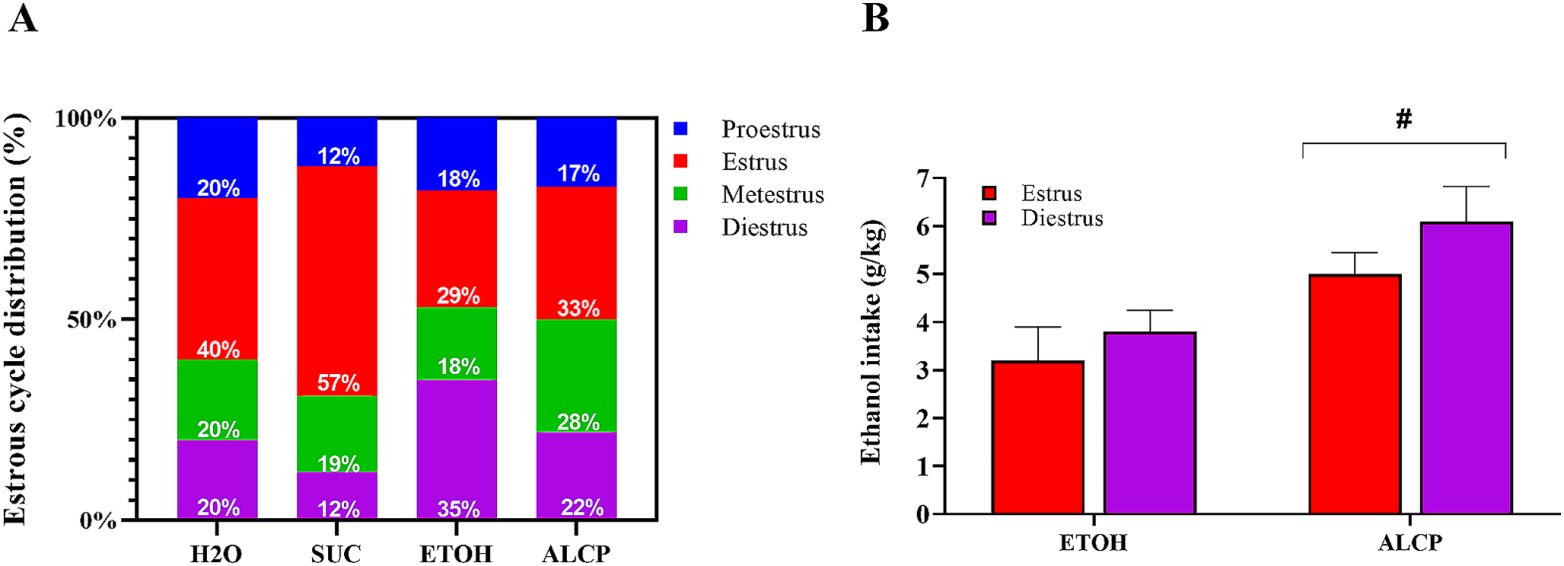
Estrous cycle during the Preference Test Day. (**A**) Estrous cycle distribution across the groups. (**B**) Comparison of ethanol intake within the cycles Estrus and Diestrus. H2O (water); SUC (sucrose); ETOH (ethanol); ALCP (alcopop). Data displayed as mean ± SEM, and significant difference when *P* < 0.05 (# compared between groups)

The estrous cycle were checked in the female rats and the percentage distribution are displayed in Fig. 6 (Fig. 6A). Analysing only ETOH and ALCP groups within Estrus or Diestrus cycles (most prevalent cycling phases after PT day), a two-way ANOVA test found no interaction of Group × cycle (*F*(1,17) = 0.185; *P* = 0.672; Fig. 6B), however ALCP female rats had greater ethanol intake compared to ETOH rats either cycling on Estrus (#*P* = 0.033) or cycling on Diestrus phase (#*P* = 0.013), indicating that there is no effect of cycling in the ethanol intake after the PT day, rather ALCP rats drank more ethanol than ETOH in general.

### Elevated plus maze

**Fig. 7:**
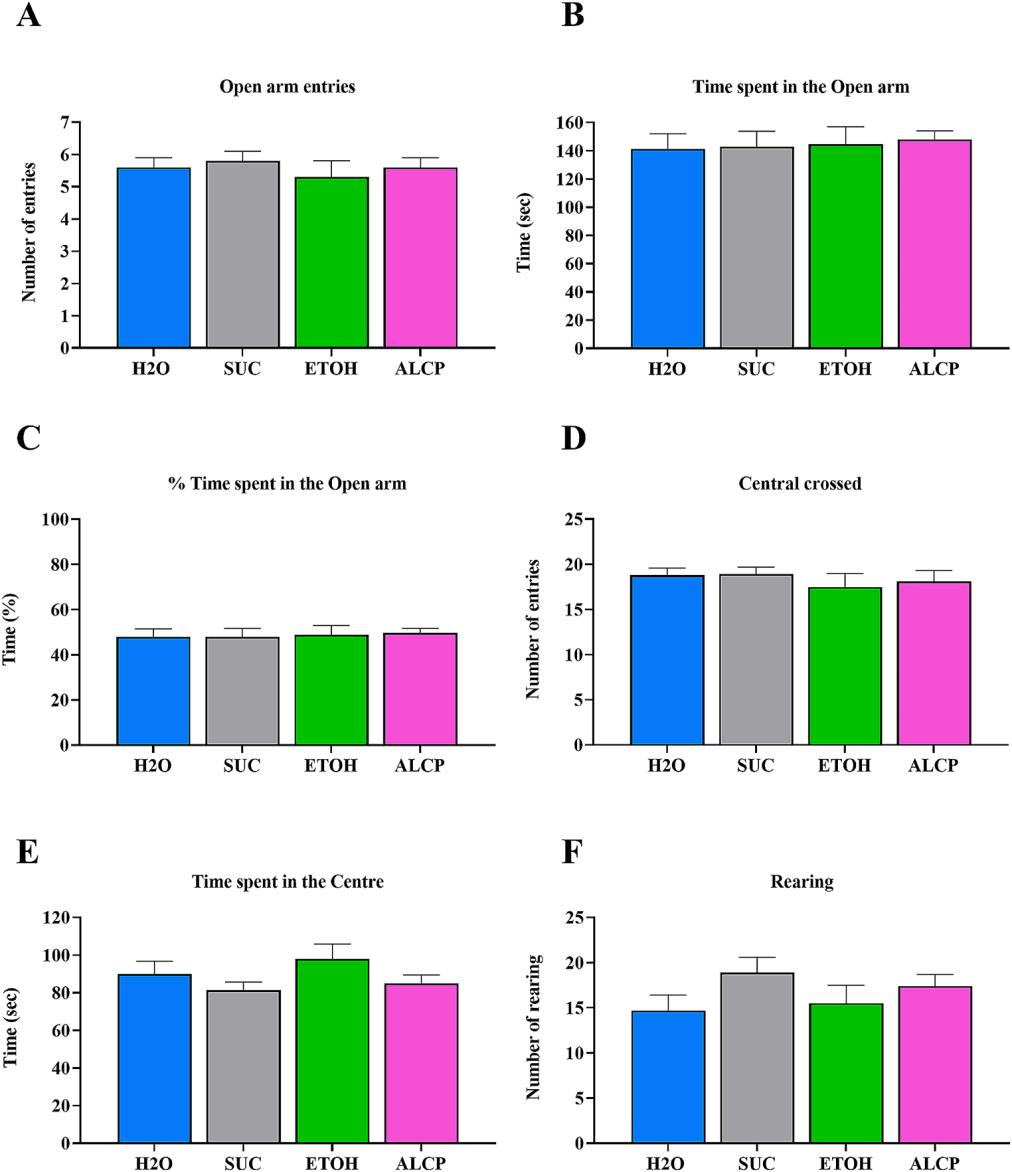
Elevated plus maze behavioural test. Behavioural manifestations evaluated in EPM test: (**A**) Open arm entries; (**B**) Time spent in open arms; (**C**) Percentage of time spent in open arms; (**D**) Total central crossed; (**E**) Time spent in the centre; (**F**) Total number of rearing in the closed arms. H_2_O, water control (blue); SUC, sucrose (grey); ETOH, ethanol (green); ALCP, alcopop (pink). Data are expressed as mean ± SEM (*n* = 12–14)

The animals underwent a 5-min EPM test for measuring anxiety-like effects of 1-week withdrawal followed chronic alcohol binge drinking. There was no significant difference revealed by 1-way ANOVA in the EPM (Fig. 7) marked by no interaction in all behaviour analysed: (A) Open arms Entries (*F*(3,49) = 0.451; *P* = 0.718); (B) Time spent on Open arms (*F*(3,49) = 0.087; *P* = 0.966); (C) percentage spent on Open arms (*F*(3,49) = 0.0708; *P* = 0.975); (D) total Central crossed (*F*(3,49) = 0.512; *P* = 0.676); (E) Time spent in the Centre (*F*(3,49) = 1.403; *P* = 0.253); and (F) Total number of Rearing in the closed arms (*F*(3,49) = 1.690; *P* = 0.181).

### Locomotor activity test

**Fig. 8:**
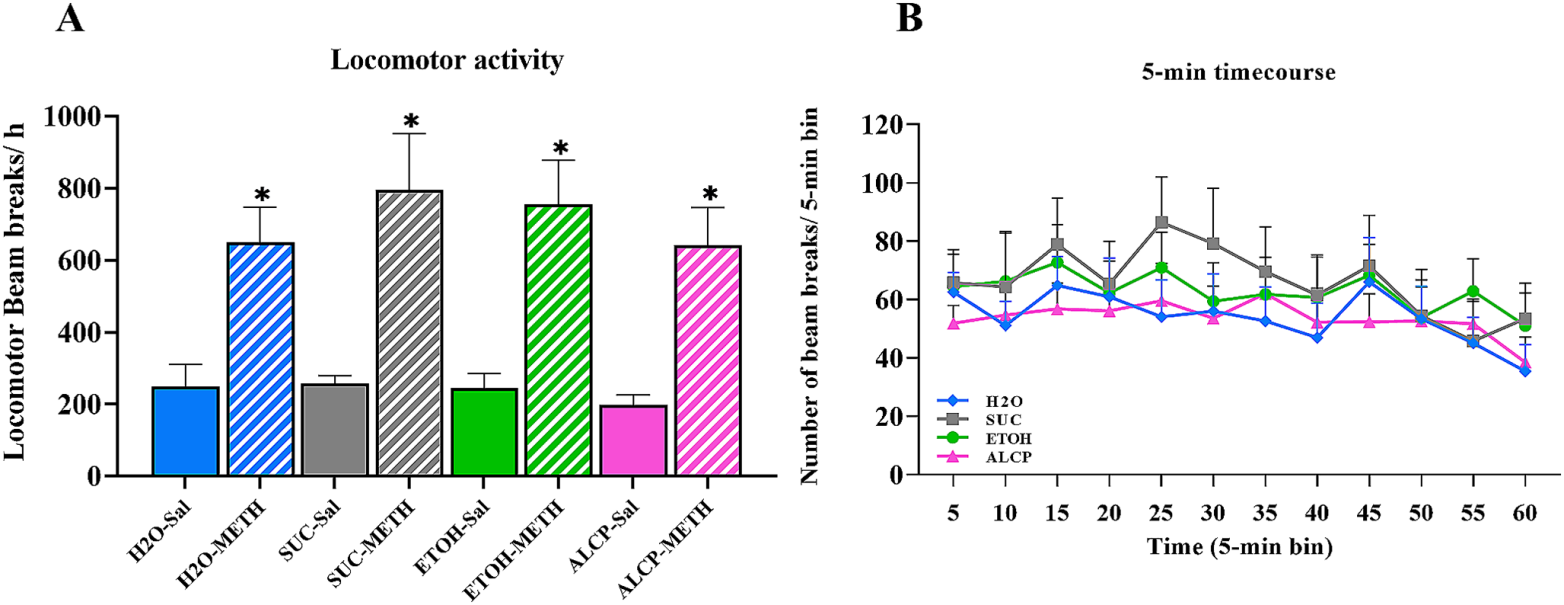
(**A**) Locomotor activity after saline or METH administration; (**B**) 5-minute time course of locomotor activity after METH administration. Sal, saline; METH, methamphetamine; H_2_O, water control (blue); SUC, sucrose (grey); ETOH, ethanol (green); ALCP, alcopop (pink). Data displayed as mean ± SEM, and significant difference when *P* < 0.05 (* compared within groups)

Locomotor activity was measured by the sum of beam breaks of the boxes (front + back + back and forth). After running a 2-way ANOVA, the 2-week alcohol withdrawal had no impact on their locomotor activity between groups after METH injection (Group × Treatment) (*F*(3,58) = 0.208; *P* = 0.891; Fig. 8). As expected, all rats which received METH injection had a significant higher locomotor activity when compared with SAL injection (*F*(1,58) = 49.236;*P < 0.001; Fig. 8A). A 5-minute time course of 1-hour locomotor activity was assessed and there were no significant differences when analysing 5-min blocks comparing across the groups (*F*(33,341) = 0.588; *P* = 0.967; Fig. 8B).

### Density of GFAP-immunoreactive astrocytes

**Fig. 9:**
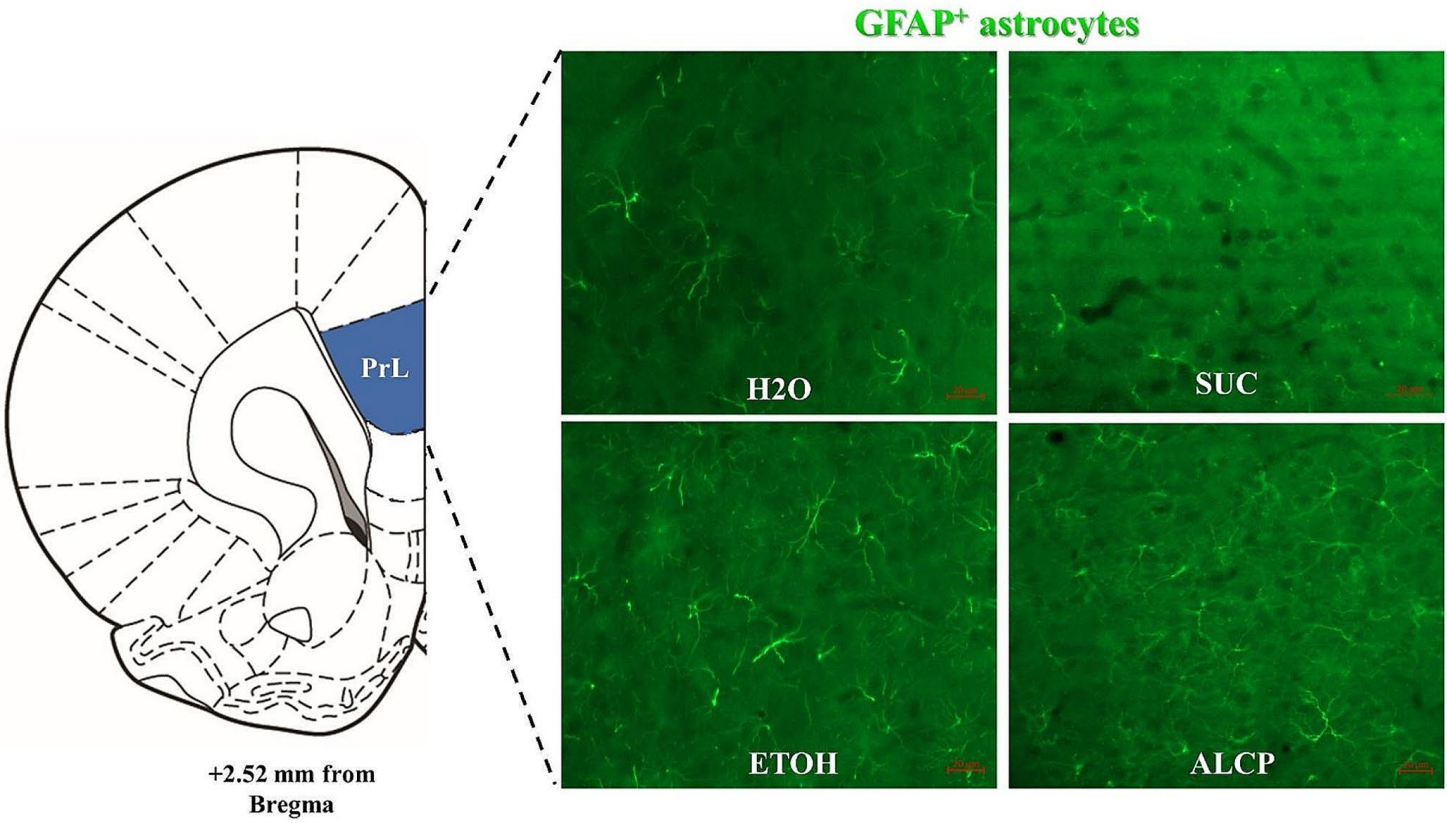
Illustrative representation of immunofluorescence staining of GFAP positive astrocytes in the prelimbic cortex (+ 2.52 mm from bregma) of female rats 2-week alcohol withdrawal. GFAP, glial fibrillary acidic protein; H_2_O, water control; SUC, sucrose; ETOH, ethanol; ALCP, alcopop. Fluorescence imagining (20x objective); staining for goat anti-GFAP with Alexa488-conjugated donkey anti-goat (green fluorescence). Scale bar: 20 μm

**Fig. 10:**
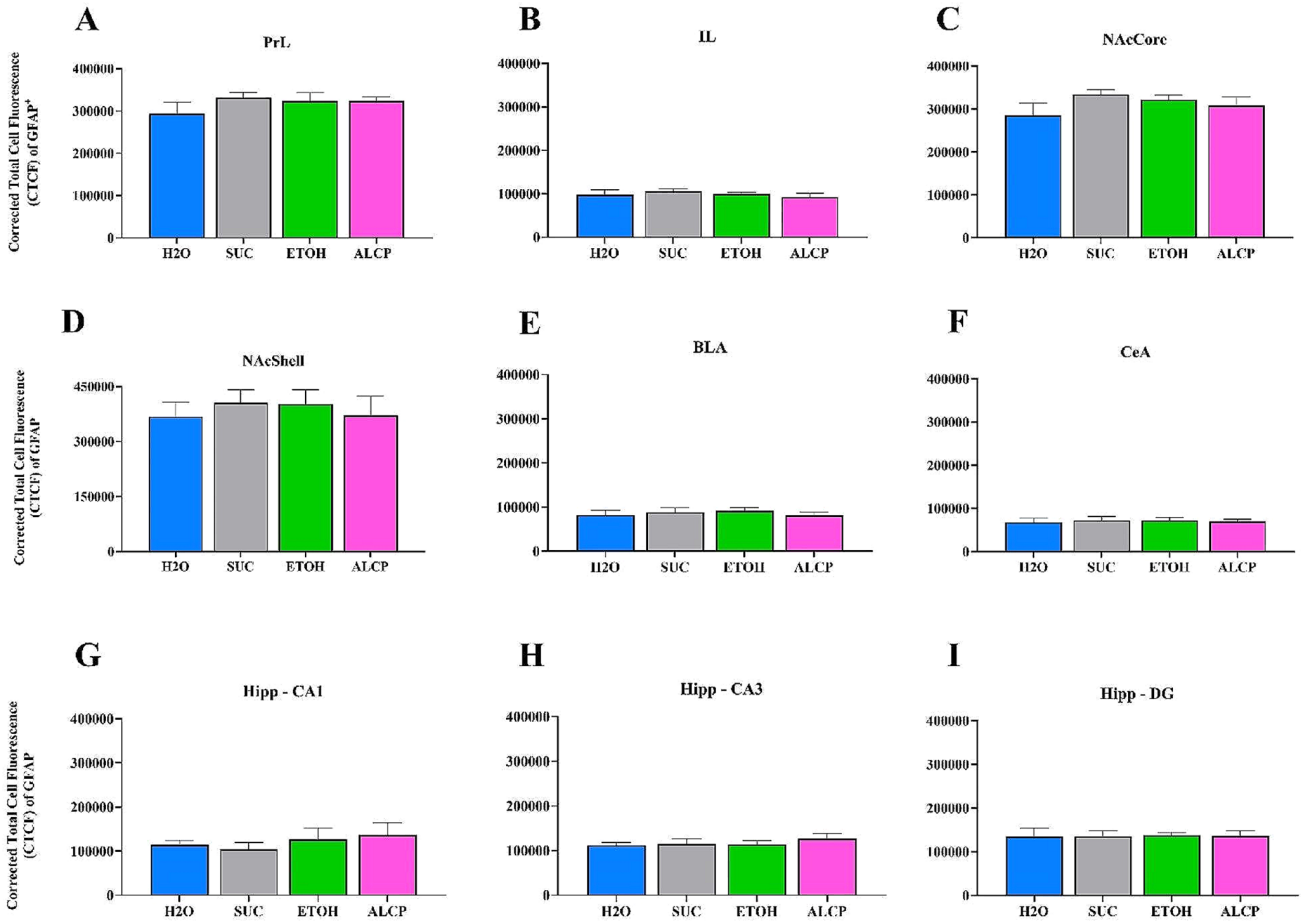
Integrated density of GFAP^+^ astrocyte expression in several brain regions. (**A**) Prelimbic; (**B**) Infralimbic; (**C**) Nucleus accumbens Core; (**D**) Nucleus accumbens Shell; (**E**) Basolateral amygdala (BLA); (**F**) Central of amygdala (CeA); Hippocampus subregions (**G**) CA-1; (**H**) CA-3; (**I**) Dendate gyrus (DG). H_2_O, water control (blue); SUC, sucrose (grey); ETOH, ethanol (green); ALCP, alcopop (pink). Data displayed as mean ± SEM

Immunofluorescence staining for GFAP astrocytes in the brain of female rats 2-week alcohol withdrawn were assessed, and Fig. 9 represents astrocyte expression only in the PrL region of all groups. There were no differences in GFAP-astrocyte density across all groups in all the brain regions analysed (Fig. 10A-I), Prelimbic (*F*(3,17) = 0.7367; *P* = 0.544), Infralimbic (*F* (3,17) = 0.404; *P* = 0.751), Nucleus Accumbens core (*F*(3,17) = 1.313; *P* = 0.302) or shell (*F*(3,17) = 0.243; *P* = 0.864), Hippocampus CA1 (*F*(3,17) = 0.468; *P* = 0.708), Hippocampus CA3 (*F*(3,17) = 0.383; *P* = 0.766) or Hippocampus DG (*F*(3,17) = 0.006; *P* = 0.999), BLA (*F*(3, 17) = 0.253; *P* = 0.857) or CeA (*F*(3,17) = 0.072; *P* = 0.973).

## Discussion

The current study sought to investigate the behavioural and brain changes in adolescent female rats exposed to an alcopop binge drinking model, including assessment of anxiety-like behaviour at 1 week of withdrawal, cross-sensitization to METH administration and astrocyte (GFAP^+^) expression in reward-related brain regions, following 2 weeks of withdrawal. ALCP rats consumed more total solution and had greater ethanol intake over time compared to the ETOH control group. Secondly, it was demonstrated that either ALCP or ETOH treatment groups preferred the low alcohol concentration (5%) over high concentration (15%) during the PT, however, when comparing the amount of solution consumed from the high dose alcohol bottle, ALCP rats had significantly greater ethanol intake when compared to ETOH rats. This preference did not appear to be influenced by the rat estrous cycle. Third, following 1-week of withdrawal from the binge drinking model, there was no significant differences in anxiety-like behaviour using the EPM test. In addition, following 2-weeks of withdrawal from the binge model, there were no differences in treatment groups on either acute METH exposure and locomotor assessment nor effect on the expression of GFAP^+^ astrocytes in the PrL, IL, NAcCore, NAcShell, Hippocampus, CeA or BLA.

Consumption of the alcopop solution in the ALCP group had an impact on female rats to increase their ethanol intake during adolescence compared to ethanol control (ETOH) animals, confirming the notion that sweetened alcoholic solutions are more palatable than ethanol solutions in adolescent females (Roberts, Heyer and Koob, 1999). Furthermore, both rewarding effects of sweet and alcohol solutions may be partially mediated by similar brain systems and may share overlapping brain mechanisms to encourage greater consumption (Kampov-Polevoy et al. 2001). This result agrees with previous studies that showed an increase of ethanol intake was achieved by using sweetened ethanol drinks (Yoneyama et al. 2008; Maldonado-Devincci et al. 2010; Broadwater et al. 2013). However, there are mixed findings in the literature, showing a reduction of ethanol intake in rats with a history of sucrose consumption during adolescence (Vendruscolo et al. 2010), or no changes between consumption levels of sweetened and unsweetened ethanol solutions (Vetter et al. 2007). However, all of these findings were reported in male rats, whereas our study only investigated female rats. To our knowledge this is the first study to demonstrate that sweetened alcoholic drinks increase ethanol intake in adolescent female rats when exposed to 1-hour daily access, which may suggest increased health risks for female consumers in the population.

In the current study we also observed that both SUC and H2O groups significantly increased their fluid consumption from day 19–34 (significance not displayed on graph, Fig. 3) greater than compared to the other groups, notably when higher concentrations of alcohol were available for consumption (day 19 onwards). It was clear from the data that SUC rats had higher overall consumption, potentially due to the taste and palatability of sucrose solution which is in line with recent theories that the rewarding effects of sugar may surpass those of addictive drugs (Alaux-Cantin et al. 2021). The higher consumption of water in the H2O group when compared to the alcohol groups is likely the result of the mild water deprivation paradigm applied in this study. When the rats were water restricted, they drank an average of approximately 6 g/day when offered a bottle of water for a 1-hour session. Several studies have also used this protocol to encourage rats to consume the aversive tasting ethanol to at least reach a minimum of binge drinking for toxic levels (Gibson et al. 2018; Hamlin et al. 2006). It is important to note that the water restriction used here did not result in differences in body weight gain in either of the groups at any point during the oral consumption paradigm.

Low concentrations of alcoholic solutions are usually chosen in numerous experiments of this type due to higher palatability for voluntary consumption in rats, especially for outbred strains of rats such as Sprague-Dawley (Rezvani et al. 2010) used in this study. The present findings showed that either ALCP or ETOH rats preferred 5% over 15% alcohol concentration during the PT day. These findings align with studies where rats were offered different alcohol concentrations and they reported higher intake of low over higher concentrations (Veale and Myers 1969). Similarly, different inbred strains of mice were tested with preference for different ethanol concentrations and most strains preferred the low dose of 3% and showed avoidance for the higher dose of 10% ethanol (Belknap et al. 1993). It is of interest, however, that although both ALCP and ETOH groups preferred the lower concentration, when analysed only within the higher concentration (15% ALCP vs. 15% ETOH), the ALCP rats consumed significantly more ethanol when compared to the ETOH rats, indicating that the presence of sucrose in alcoholic beverages can positively stimulate greater amounts of ethanol consumption in highly concentrated alcoholic products (Yoneyama et al. 2008) and may be considered risky to enable consumption of toxic amounts, particularly by girls who binge daily. Finally, in the current study and during the PT, it was shown that the phases of estrous cycle examined did not impact on the ethanol intake between groups of either low or high alcohol concentration, in line with other researchers (Priddy et al. 2017).

The behavioural manifestations during alcohol withdrawal in animals are generally anxiety- and depression-like behaviour, hypoactivity, stress, among others; signs that may resemble affective symptoms in humans (Doremus and Spear 2007). Anxiety- and affective-like behaviours are classically measured in preclinical research through the EPM test, allowing the subjects to explore the apparatus and in general, the animals tend to stay in perceived safe areas such as the closed arms than in the more aversive areas like the open arms of the EPM (Walf and Frye 2007). After 1 week of alcohol withdrawal, we found no significant effects in several anxiety-like behaviours evaluated in the EPM by any of the groups, suggesting that either binge exposure of sucrose or alcohol alone or in combination (ALCP) during adolescence did not impact on the exploratory and anxiety-like behaviours at early adulthood. Decades of research into alcohol use has determined that withdrawal from use manifests in many different behaviours and are dependent on timing, dose, age and strain of the rat (Doremus and Spear 2007; Costa et al. 2015; Logan et al. 2012). Prior research in adolescence alcohol binge drinking exposure increased the exploration behaviour in the open arm after 1 month withdrawal during adulthood in male rats compared to the controls (Gilpin et al. 2012), while here we showed that female rats explored the open arm similarly across groups following 1 week of withdrawal. Open arm was explored approximately ~ 48% by all rats regardless of their previous treatment in the present study, whereas the reduction to 40% seen in anxiety-like effects in the EPM occurred after 1-month of withdrawal (Gilpin et al. 2012), indicating that a longer period without alcohol access may show some changes in anxiety-like behaviour that were not detected here.

Repeated exposure to psychostimulant drugs results in an enhanced behavioural response to that stimulus following a period of abstinence causing a phenomenon called behavioural sensitization, and is manifested in rodents as an increase of locomotor activity (Pierce and Kalivas 1997; Kalivas et al. 1998). Cross-sensitization occurs when a novel drug produces an enhanced behavioural response in animals previously treated repeatedly with a different drug, suggesting shared neural mechanisms and changes in the brain (Meyer and Phillips 2007). In the current study, we tested the acute effects of METH administration within 2-weeks of binge withdrawn rats and found no significant changes in locomotor activity across the groups. Similarly to our findings, Fultz and Szumlinski (2018) did not observe any acute effect in METH-induced locomotor activity in male mice with prior binge drinking history across four different METH doses, however an experiment testing the same protocol using only female mice with a history of alcohol drinking identified that a lower METH dose injected (0.25 mg/kg ip) was sufficient to induce place-preference in the conditioned place preference behavioural test (Sern et al. 2020). A previous study has also shown that cross-sensitization occurred in ethanol-sensitized rats when they exhibited an increased response to cocaine challenge (Xu and Kang 2019), although it was measured 24 h after the last ethanol administration with no withdrawal period. Another study revealed cross sensitization between ethanol-sensitized mice challenged with METH but only at a high dose of METH (5 mg/kg), not seen at a lower dose of METH used in the current study, although noting the different species used (Tschumi et al. 2020). It is possible that 2 weeks of withdrawal used in the current study may be considered as an early time point for measuring cross-sensitisation behavioural effects. It is often shown that enduring differences in neural circuits and dopamine responses require sensitization protocols for psychostimulants that have a withdrawal period of at least 14–21 days in rats (Wearne et al. 2015; Umpierrez et al. 2022). For example, Zapata et al. (2006) observed that sensitization to ethanol occurred after 14 days withdrawal in male mice. Furthermore, METH acts directly on the dopaminergic system whereas alcohol exerts indirect actions (Boileau et al. 2003) which may impact on how quickly and by what mechanisms sensitization may occur between drug types. For example, researchers have identified that basal NAc dopamine neurotransmission altered by ethanol was transient and was normalized after day 1 of withdrawal in ethanol-sensitized mice, suggesting the ability for ethanol to express behavioural sensitization with no overall baseline change in dopamine in the NAc (Zapata et al. 2006).

Surprisingly, the 2-week period of alcohol withdrawal after a chronic alcohol binge drinking model applied here with female rats did not alter the density of GFAP^+^ labelled astrocytes in several reward related brain regions: PrL, IL, NAcCore, NAcShell, hippocampus, CeA or BLA. This study seems to be the first to investigate the chronic effects of adolescent alcohol binge drinking on astrocyte regulation in different brain regions of young adult rats. According to Risher and colleagues (2015), no morphological signs of astrocyte reactivity in the CA1 region of the hippocampus was detected after alcohol withdrawal in adulthood. Using western blot techniques, an increase in the levels of GFAP was detected in hippocampus and hypothalamus, but not seen in the PFC of male rats which received chronic ethanol administration via gavage (Villavicencio-Tejo et al. 2021). A study with more aggressive amounts of ethanol intake (~ 9 g/kg/day) showed an upregulation of vimentin, another protein expressed by astrocytes, only after a short period of alcohol withdrawal (4–7 days) reporting that it returned to control levels after a 14-day withdrawal in the hippocampus (Kelso et al. 2011). Accordingly, they also showed cell death beginning when the animals were still intoxicated and persisted for a week, suggesting potential recovery of hippocampal cells, and the reactive astrocytes may contribute to this repair. This current study revealed a moderate to high ethanol intake levels overtime, ranging from 1.1 to 3.4 ± 0.3 g/kg/h (with the highest amount by ALCP female rats), an amount sufficient to cause binge intoxication (Holgate et al. 2017), however no significant changes in astrocytes were detected after 2-weeks of withdrawal.

Unexpectedly, there was no GFAP astrocyte density change in the NAcCore, as Bull and colleagues (2014) demonstrated that at 3 weeks abstinence from ethanol the expression of astrocytes increased in this region. In addition, we did not observe any alteration on GFAP-positive astrocytes in the PrL, as reported by a few studies as a decrease (Miguel-Hidalgo 2005) or an increase (Dalçik et al. 2009; Udomuksorn et al. 2011). Importantly, in parallel with the GFAP marker, it would be interesting to investigate other neurotransmitter measures such as for glutamate staining (e.g. vesicular glutamate transporter; vGluT1), as astrocytes regulate glutamatergic homeostasis implicated in alcohol abuse (Adermark and Bowers 2016) as well as co-express vGluT1 in their processes (Verkhratsky et al. 2016).

Given the variability of alcohol exposure and withdrawal methodologies and their results in the literature, methodological limitations are an important consideration for this current investigation. The model of alcohol binge drinking in adolescent female rats used in this study produced moderate ethanol intake levels, however comparable to cause sufficient binge intoxication (Holgate et al. 2017), although we did not measure blood ethanol concentrations to verify BAC. Indeed, using a paradigm that ensured binge intoxication by BAC measure could potentially show effects on anxiety-like behaviour after 1 week of withdrawal as applied in this study, and possibly more behavioural tests could be explored such as open field, social interaction and light and dark box. Alternatively, one week of withdrawal may have been too limited to detect anxiety effects produced by binge drinking, which may be highest later on in the withdrawal period, as seen by others with longer periods (Gilpin et al. 2012). Another potential confound for the cross-sensitization results could be that the acute test with METH occurred too early and may not have allowed time for enduring dopamine changes to enable the expression of sensitization. Experiments designed for measuring drug sensitization start analysing the ambulation effect at least after 14–21 days of withdrawal (Wearne et al. 2015; Umpierrez et al. 2022), allowing the neural adaptations to occur, as many transient changes occur in the first week of withdrawal that may produce mixed behavioural and neurobiological results at this time point (Pierce and Kalivas 1997). Lastly, with the limited literature regarding alcohol binge drinking and astrocyte levels or function, it would be ideal to measure at an earlier withdrawal period than used in this study, such as at 1-week point used by Gomez and colleagues (2018), to identify and compare whether the alcohol and sucrose amounts taken by the rats would increase astrocyte activity in these brain regions, especially in the PrL, NAc and hippocampus, where the GFAP^+^ astrocytes densities were more highly expressed. Future directions could also explore morphological changes using sholl analysis as a single-cell technique to investigate changes in the astrocytes ramifications, although it would require 3-dimensional images as well as a high-magnification confocal microscopy images for better results.

The alcopop binge drinking model applied in this experiment was able to increase daily ethanol intake, in which the addition of sucrose in the alcoholic solution encouraged the increase of alcohol consumption more than with ethanol alone, in adolescent female rats. However, rats preferred the low alcohol levels and this behaviour was not dependent on estrous cycle. These behaviour effects and subsequent withdrawal were not associated with changes to anxiety-like or METH induced behaviours at the times tested. In addition, it was demonstrated that long period of withdrawal had no impact on the expression of GFAP in astrocytes in many reward-related brain regions, suggesting that any astrocyte changes may be fully recovered after 2-weeks of alcohol withdrawal in this model. Future studies should investigate different timings of withdrawal to fully understand the impact on behaviour and brain of alcohol binge-drinking in young adolescent females.
